# Occurrence of 2 Uncommon Findings in a Patient With Immunoglobulin G4–Related Disease: Maxillary Sinus Involvement and AA (Amyloid A) Amyloidosis

**DOI:** 10.5152/ArchRheumatol.2025.11132

**Published:** 2025-09-01

**Authors:** Melih Kızıltepe, Emel Oğuz Kökoğlu, Hüseyin Kaplan, Tuğba Kahraman Denizhan, Celil Barlas Cengiz, Sevil Kocadağ, Hülya Akgün, Abdurrahman Soner Şenel

**Affiliations:** 1 Division of Rheumatology, Department of Internal Medicine, Erciyes University Faculty of Medicine, Kayseri, Türkiye; 2 Division of Rheumatology, Department of Physical Medicine and Rehabilitation, Erciyes University Faculty of Medicine, Kayseri, Türkiye; 3 Department of Pathology, Erciyes University Faculty of Medicine, Kayseri, Türkiye

Dear Editor,

A 68-year-old female patient, whom provided informed consent, experienced progressive weakness, fatigue, weight loss, dyspnea, and cough over 2 years. During this period, she was treated several times for pneumonia but experienced recurrent elevations of acute-phase reactants (APRs) despite the use of antibiotics. She presented to the dental clinic with gingival swelling and pain. Radiological and clinical examination revealed a mass measuring approximately 3 cm extending from the base of the maxillary sinus to the oral cavity (Figure 1A and B). The biopsy, initially suspected of malignancy, revealed markedly high levels of plasma cells immunostained for IgG and IgG4, with an IgG4:IgG ratio of 25%-30%, suggesting IgG4-related disease (IgG4-RD) (Figure 1C-F). The patient was referred to our hospital. The rheumatological examination confirmed ongoing constitutional symptoms. Laboratory results showed an erythrocyte sedimentation rate (ESR) of 135 mm/h (0-20), C-reactive protein (CRP) level of 179 mg/L (0-5), complement C4 level of 43 mg/dL (0-40), and complement C3 level of 178 mg/dL (90-180). The IgG and IgG4 levels were 2508 (700-1600) mg/dL and 0.59 (0.03-2) g/L, respectively. Autoantibodies, including rheumatoid factors, anticyclic citrullinated peptide antibodies, antinuclear antibodies, and antineutrophil cytoplasmic antibodies, were negative. The renal function tests and urinary microprotein-to-creatinine ratio were within normal limits. Serum protein electrophoresis showed polyclonal hypergammaglobulinemia. Echocardiography and infection screenings were performed to rule out other causes. Malignancy was ruled out by positron emission tomography/computed tomography. Based on the clinical, radiological, and histopathological findings, the patient was diagnosed with IgG4-RD and treated with methylprednisolone (1 mg/kg) and methotrexate (15 mg/week). The APRs decreased rapidly with clinical improvement. In retrospect, the diagnosis was made in accordance with the 2019 ACR (American college of rheumatology)/EULAR (European league against rheumatism) Classification Criteria, based on the presence of a tumefactive lesion in the maxillary sinus on imaging, systemic inflammation with markedly elevated APRs, dense lymphoplasmacytic infiltration on histology, immunostaining results (an IgG4:IgG ratio of 0%-40% and >10 IgG4+ plasma cells per high-power field), consistent clinical findings, a rapid clinical and biochemical response to treatment, and the exclusion of other mimickers.

However, during steroid tapering in the 4th month, proteinuria developed (4.7 g/day), and ESR (120 mm/h) and CRP (124 mg/L) levels increased again. The creatinine level was normal. The methylprednisolone dose was increased to 1 mg/kg, and a renal biopsy was performed, which revealed renal AA amyloidosis (Figure 1G and H). Familial Mediterranean fever was ruled out. The existing findings confirmed the diagnosis of secondary AA amyloidosis owing to IgG4-RD. Rituximab (1 g) was administered on days 1 and 15. Six months after treatment, the CRP (3.78 mg/L), proteinuria (0.2 g/day), and IgG levels (970 mg/dL) were all within the normal ranges.

Immunoglobulin G4–related disease is an uncommon fibroinflammatory condition. The disease may present with a painless mass. Immunoglobulin G4–related disease predominantly affects the retroperitoneum, pancreas, aorta, lacrimal, salivary, and thyroid glands; however, it can affect any body structure.^1^ The involvement of the paranasal sinus is relatively rare.^2^ Our patient presented with a mass of approximately 3 cm that extended from the maxillary sinus to the oral cavity.

The immunopathogenesis of IgG4-RD is not clearly understood. While histopathological assessment is central to diagnosis, clinical, radiological, and serological findings are also critical—particularly in the sinonasal region, where several conditions such as lymphoma, granulomatosis with polyangiitis, fungal infections, and chronic rhinosinusitis can mimic IgG4-RD.^3^ In our case, these potential mimickers were systematically ruled out through a combination of histological analysis, serological and microbiological testing, imaging, and clinical evaluation. Although storiform fibrosis and obliterative phlebitis were not observed, these features are known to vary across different organs and may be absent.

The evaluation of the electronic hospital records revealed that the patient had elevated APRs for approximately 7 years. Although the patient initially responded to steroid treatment, she experienced subsequent proteinuria and APR reelevation. The patient’s long-standing inflammatory state likely contributed to the onset of amyloidosis. AA amyloidosis is a very rare complication of IgG4-RD, with only six cases reported in the literature. In these cases, the interval between IgG4-RD manifestation and amyloidosis diagnosis ranged from 5 months to 20 years,^4^ and the patients were treated with high-dose systemic steroids and anti-CD20 monoclonal antibodies.^4-6^ In our case, the disease flare and AA amyloidosis that developed during methotrexate treatment were successfully treated with rituximab. Other causes of chronic inflammation were thoroughly excluded, including infections, malignancy, and autoinflammatory syndromes. Given the absence of alternative etiologies, IgG4-RD was considered the most likely underlying cause of AA amyloidosis.

In conclusion, IgG4-RD should be suspected when a mass is observed in an unusual location, such as the paranasal sinus. Despite its rarity, the fact that long-term inflammation in IgG4-RD can lead to AA amyloidosis should be considered. This case report highlights the possible association between two rare conditions and IgG4-RD.

## Figures and Tables

**Figure 1.**
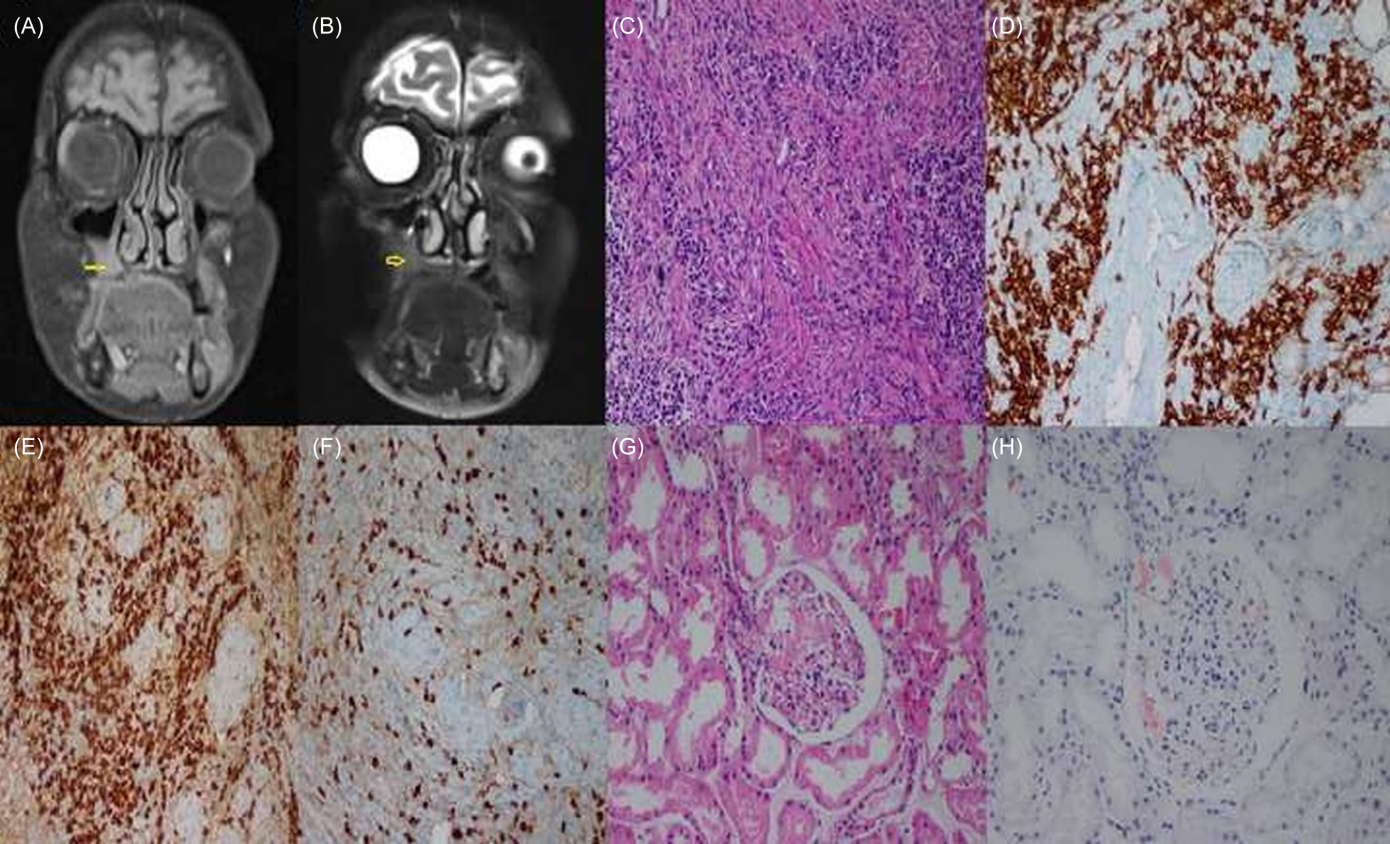
Imaging and histological samples from the patient. Radiological and mass/renal biopsy samples from a 68-year-old female patient diagnosed with IgG4-RD. (A) T1 hyperintense and (B) T2 mildly hyperintense MR images of an irregularly defined mass measuring 25 × 14 mm and extending from the right maxillary sinus floor to the oral cavity (yellow arrows); (C) Hematoxylin-and-eosin sections showing plasma-cell-rich mononuclear inflammatory cell infiltration on a fibrotic background (×200); (D) Immunohistochemistry stain showing intense CD38 positivity in inflammatory cells (×200); (E) Immunohistochemistry stain showing intense IgG positivity in inflammatory cells (×200). (F) A sample obtained from the patient’s mass underwent immunohistochemical staining for IgG4, with a ratio of IgG4-positive plasma cells of 0.25-0.30; (G) Hematoxylin-and-eosin sections showing an accumulation of a homogeneous amorphous substance in the glomeruli, expanding the mesangial areas (×200); (H) histochemistry stain showing brick-colored staining with Congo-red (×200). CD38, cluster of differentiation 38; IgG4-RD, immunoglobulin G4-related disease; MR, magnetic resonance.

## Data Availability

The data that support the findings of this study are available on request from the corresponding author.
